# Investigations of the Kinetics and Mechanism of Reduction of a Carboplatin Pt(IV) Prodrug by the Major Small-Molecule Reductants in Human Plasma

**DOI:** 10.3390/ijms20225660

**Published:** 2019-11-12

**Authors:** Yang Liu, Hongwu Tian, Liyao Xu, Li Zhou, Jinhu Wang, Benyan Xu, Chunli Liu, Lars I. Elding, Tiesheng Shi

**Affiliations:** 1 College of Chemistry, Chemical Engineering and Materials Science, Zaozhuang University, Zaozhuang 277160, China; chemliuyang@uzz.edu.cn (Y.L.); zhouli@uzz.edu.cn (L.Z.); 101346@uzz.edu.cn (B.X.); 2 College of Chemistry and Environmental Science, and the MOE Key Laboratory of Medicinal Chemistry and Molecular Diagnostics, Hebei University, Baoding 071002, China; thw9125@163.com (H.T.); mcyao82@163.com (L.X.); 3 Center for Analysis and Synthesis, Department of Chemistry, Lund University, P.O. Box 124, SE 221 00 Lund, Sweden

**Keywords:** carboplatin, Pt(IV) prodrug, human plasma, reduction, kinetic analysis, mechanism

## Abstract

The development of Pt(IV) anticancer prodrugs to overcome the detrimental side effects of Pt(II)-based anticancer drugs is of current interest. The kinetics and reaction mechanisms of the reductive activation of the carboplatin Pt(IV) prodrug *cis,trans*-[Pt(cbdca)(NH_3_)_2_Cl_2_] (cbdca = cyclobutane-1,1-dicarboxylate) by the major small-molecule reductants in human plasma were analyzed in this work. The reductants included ascorbate (Asc), the thiol-containing molecules L-cysteine (Cys), DL-homocysteine (Hcy), and glutathione (GSH), and the dipeptide Cys–Gly. Overall second-order kinetics were established in all cases. At the physiological pH of 7.4, the observed second-order rate constants *k*′ followed the order Asc << Cys–Gly ~ Hcy < GSH < Cys. This reactivity order together with the abundances of the reductants in human plasma indicated Cys as the major small-molecule reductant in vivo, followed by GSH and ascorbate, whereas Hcy is much less important. In the cases of Cys and GSH, detailed reaction mechanisms and the reactivity of the various protolytic species at physiological pH were derived. The rate constants of the rate-determining steps were evaluated, allowing the construction of reactivity-versus-pH distribution diagrams for Cys and GSH. The diagrams unraveled that species **III** of Cys (^−^SCH_2_CH(NH_3_^+^)COO^−^) and species **IV** of GSH (^−^OOCCH(NH_3_^+^)CH_2_CH_2_CONHCH(CH_2_S^−^)- CONHCH_2_COO^−^) were exclusively dominant in the reduction process. These two species are anticipated to be of pivotal importance in the reduction of other types of Pt(IV) prodrugs as well.

## 1. Introduction

In cancer chemotherapy, Pt(II)-based drugs, including cisplatin, carboplatin, and oxaliplatin, have been and are still very important and are used world-widely [1,2,3,4]. However, in spite of the tremendous success of these drugs, they also give rise to mild or severe side effects [5], such as nephrotoxicity, ototoxicity, and gastrointestinal toxicity for cisplatin [5,6], hematological toxicity and severe myelo-suppression for carboplatin [5,6], and severe neurotoxicity for oxaliplatin [5,6]. The two major approaches to overcome or minimize these detrimental side effects have been *(a)* the development of “rescuing agents” to be used together with the Pt(II) drugs [7,8] and *(b)* the conversion of the Pt(II) drugs into their Pt(IV) derivatives, assuming that these derivatives can be delivered to tumor sites more efficiently and with less harmful side effects [9,10,11,12,13,14,15,16,17,18,19,20]. Whereas the first approach has had a very limited success [7,8], the second one is still of current interest [9,10,11,12,13,14,15,16,17,18,19,20].

Compared to the square-planar, substitution-labile Pt(II)-based anticancer drugs, Pt(IV) anticancer active compounds are octahedrally coordinated and virtually substitution-inert. This should result in a longer lifetime under physiological conditions. These Pt(IV) compounds are commonly regarded as prodrugs, since their reduced products (often their Pt(II) counterparts) are the real anticancer species [1,2,3,4,9,10,11,12]; the reduction leads, in fact, to their activation. A large number of Pt(IV) prodrugs derived from cisplatin, carboplatin, and oxaliplatin have been exploited [9,10,11,12,13,14,15,16,17,18,19,20]. Despite this huge effort, the exact control of prodrug activation remains unresolved [21], due partly to the fact that there are so many strong reductants in biological systems. When Pt(IV) prodrugs are administered intravenously, as most Pt-based drugs are [6], they come immediately in contact with human blood. Small-molecule reductants of high abundance in human plasma include ascorbic acid, l-cysteine (Cys), DL-homocysteine (Hcy), glutathione (GSH), and the dipeptide Cys–Gly [22]. They might all be of critical importance for the activation of Pt(IV) prodrugs [23].

**Figure 1:**
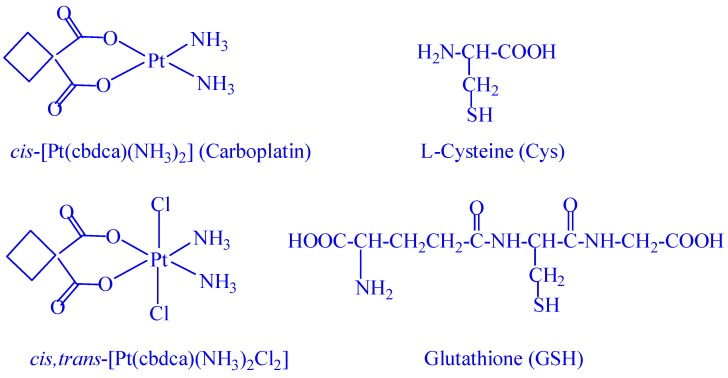
Structures of carboplatin, its prodrug *cis,trans*-[Pt(cbdca)(NH_3_)_2_Cl_2_], and of l-cysteine (Cys) and glutathione (GSH) in their neutral forms.

Previous studies by different research groups have demonstrated that large molecules in the plasma such as human serum albumin, hemoglobin, and cytochrome c might also be of critical importance for the activation of Pt(IV) prodrugs under certain circumstances [24,25,26,27]. Regarding the complicated matrix of human plasma [22], some sophistical instrumental methods have recently been developed, in particular by the Keppler and Hambley groups, for the investigation of the activation processes when different Pt(IV) prodrugs are put into the matrix [27,28,29,30,31]. However, the major small-molecule reductants ascorbic acid, Cys, and GSH are commonly used for estimation of the activation processes of various Pt(IV) prodrugs [12,14,15,16,19,20]. Kinetic and mechanistic studies to elucidate the detailed reduction processes by these small-molecule reductants are still of current interest [23,32,33,34]. We have analyzed in detail the redox kinetics of one of the carboplatin Pt(IV) prodrugs, *cis,trans*-[Pt(cbdca)(NH_3_)_2_Cl_2_] (cbdca = cyclobutane-1,1-dicarboxylate) [17,18], by these major small-molecule reductants in human plasma by stopped-flow spectrophotometry and identified the most reactive protolytic species at the physiological pH of 7.4. The structures of carboplatin, its Pt(IV) prodrug *cis,trans*-[Pt(cbdca)(NH_3_)_2_Cl_2_], Cys, and GSH are shown in Figure 1.

*Trans*-dichlorido-Pt(IV) compounds are still an option in the design of new Pt(IV) prodrugs [17,18,35], albeit loosing the spotlights due to their quick reductions. In this work, our focus is on the kinetics and detailed mechanistic analysis of the fast reduction processes, trying to estimate its approximate surviving time in the plasma and establish if the prodrug can reach the target DNA molecules before reduction.

## 2. Experimental

### 2.1. Reagents and Solutions

GSH, Cys, Hcy, and monosodium ascorbate were obtained from Sigma-Aldrich (St. Louis, MO). The dipeptide Cys–Gly (purity >95%) was purchased from Dgpeptide Co., Ltd (Hongzhou, China). The prodrug *cis,trans*-[Pt(cbdca)(NH_3_)_2_Cl_2_] (cbdca = cyclobutane-1,1-dicarboxylate) was from a batch synthesized and characterized recently [36]. Acetic acid, sodium acetate, sodium chloride, hydrochloric acid, phosphoric acid, sodium dihydrogen phosphate, disodium hydrogen phosphate, sodium bicarbonate, sodium carbonate, trisodium phosphate, and sodium perchlorate were all obtained in their purest forms available either from Alfa Aesar (Shanghai, China) or from Fisher Scientific (Shanghai, China) and were used without further purification. For pH meter calibrations, standard buffers at pH 4.00, 7.00, and 10.00 were purchased from Fisher Scientific. Doubly distilled water was used to prepare all solutions. Buffers with pH between 2.47 and 11.24 and an ionic strength (*μ*) of 1.0 M were prepared as described previously [23,36]. 

### 2.2. Measurements of Kinetic Data

Stock solutions of thiols were prepared by adding the desired amount of thiol to a buffer solution of specific pH, which was then flushed for 10 min with nitrogen. Stock solutions of 1.0 mM [Pt(cbdca)(NH_3_)_2_Cl_2_] were prepared by dissolving the desired amount of the Pt(IV) complex in a solution containing 0.90 M NaClO_4_, 0.09 M NaCl, and 0.01 M HCl. These stock solutions were only used for about 2 h. Solutions of Pt(IV) and a thiol for kinetic measurements were prepared by dilution of the abovementioned stock solutions with the same pH buffer and then flushed for 10 min with nitrogen before loading onto the stopped-flow instrument. Kinetic traces were run by mixing equal volumes of the Pt(IV) and thiol solutions directly in an Applied Photophysics SX-20 stopped-flow spectrometer (Applied Photophysics Ltd., Leatherhead, U.K.). All kinetic runs were carried out under pseudo first-order conditions with [thiol]_tot_ ≥ 10·[Pt(IV)], where [thiol]_tot_ denotes the total concentration of the thiols. Kinetic traces were monitored in the region of 250–280 nm, where the absorbance decreased due to the reduction of [Pt(cbdca)(NH_3_)_2_Cl_2_]. In the case of ascorbate, however, these pseudo first-order conditions could not be employed because of the high absorbance background of ascorbate in the wavelength region. Therefore, this reaction was followed at 265 nm in a pH 7.40 buffer with the stopped-flow instrument and [Pt(IV)] = [Asc]_tot_, where [Asc]_tot_ denotes the total concentration of ascorbate. Stock solutions of ascorbate were only used for less than 20 min (vide infra). 

### 2.3. Product Analysis

High-resolution mass spectra were recorded on an LC/Q-Orbitrap mass spectrometer with a heated electrospray ionization source (ESI) in positive mode (Thermo Scientific, Bremen, Germany). Samples of a fresh solution of 8 mM Cys in 10 mM HAc and a reaction mixture containing 8 mM Cys and 1 mM [Pt(cdbca)(NH_3_)_2_Cl_2_] in 10 mM HAc after a reaction time of about 5 min were run.

## 3. Results and Discussion

### 3.1. Reduction of [Pt(cdbca)(NH_3_)_2_Cl_2_] by Biothiols

**Figure 2:**
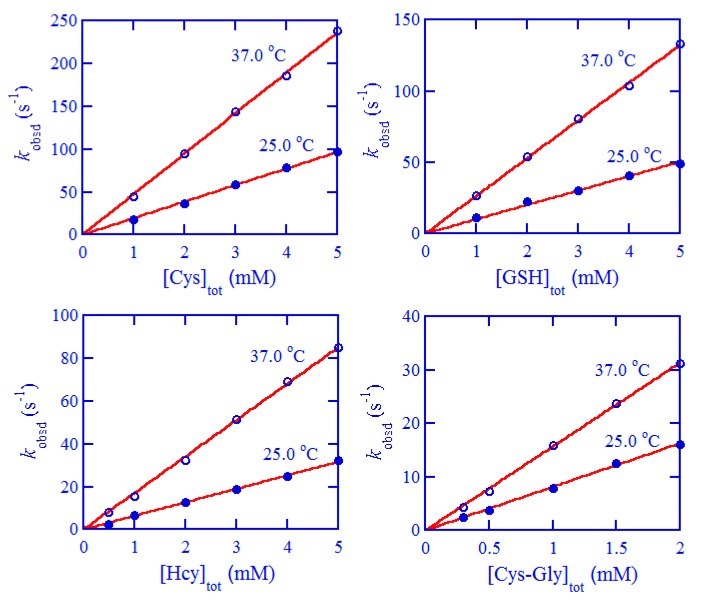
Plots of *k*_obsd_ versus total concentration of thiols at 25.0 and 37.0 °C and ionic strength (*μ*) = 1.0 M. Hcy: DL-homocysteine.

Under pseudo first-order conditions, all the kinetic traces for the reduction of [Pt(cbdca)(NH_3_)_2_Cl_2_] were well simulated by single exponentials, confirming that the reduction is indeed first-order in [Pt(IV)]; pseudo first-order rate constants *k*_obsd_ were obtained from these simulations and are reported in this work as average values from 3–7 duplicate runs. Plots of *k*_obsd_ versus [thiol]_tot_ are displayed in Figure 2 at the physiological pH (pH 7.40) and in Figures S1 and S2 as a function of pH; unambiguously, these plots are all linear and passing through the origin. Hence, the reduction is also first-order in [thiol]_tot_, and the overall second-order kinetics is expressed by Equation (1), where *k*′ stands for the observed second-order rate constants. Values of *k*′ evaluated at pH 7.40 for the four biothiols are provided in Table 1, whereas values of *k*′ as a function of pH are summarized in Table S1 in Supplementary Materials for Cys and GSH.

−d[Pt(IV)]/dt = *k*_obsd_[Pt(IV)] = *k*′[thiol]_tot_[Pt(IV)] (1)

### 3.2. Reduction of [Pt(cdbca)(NH_3_)_2_Cl_2_] by Ascorbate

**Figure 3:**
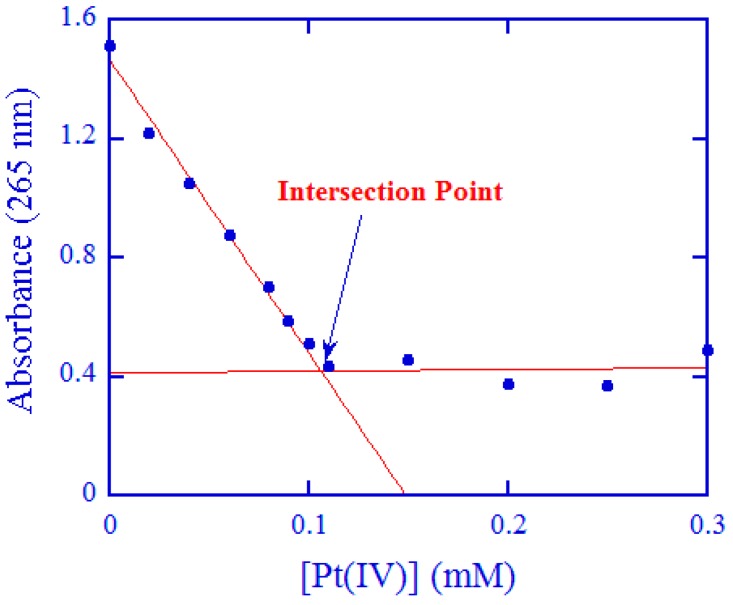
Spectrophotometric titrations for the determination of the redox stoichiometry. [Asc]_tot_ = 0.10 mM was kept constant, and [Pt(IV)] was changed from 0 to 0.30 mM. The reaction medium was pH 7.40 phosphate buffer, and the ionic strength *μ* = 1.0 M. The reaction time for each of the reaction mixtures was 4–5 min.

The stoichiometry of the reaction between [Pt(cdbca)(NH_3_)_2_Cl_2_] and ascorbate was studied by the spectrophotometric titration method described earlier [23,37,38,39]. A series of reaction mixtures were prepared in a phosphate buffer at pH 7.40 with [Asc]_tot_ = 0.10 mM kept constant, whereas [Pt(IV)] was varied between 0 and 0.30 mM. The absorbance at 265 nm was measured after a reaction time of 4–5 min for each of the reaction mixtures; electronic spectra and absorbance values were recorded by an UV–vis spectrophotometer (TU-1900, Beijing Puxi, Inc., Beijing, China). The absorbance as a function of [Pt(IV)] is shown in Figure 3. Unequivocally, the data points can be trailed by two crossing straight lines, and the intersection point imparts a ratio ∆[Asc]_tot_/∆[Pt(IV)] = 1/(1.06 ± 0.05). This ratio indicates a 1:1 stoichiometry within experimental errors, suggesting that ascorbate is oxidized to dehydro-ascorbate [23,40,41].

**Figure 4:**
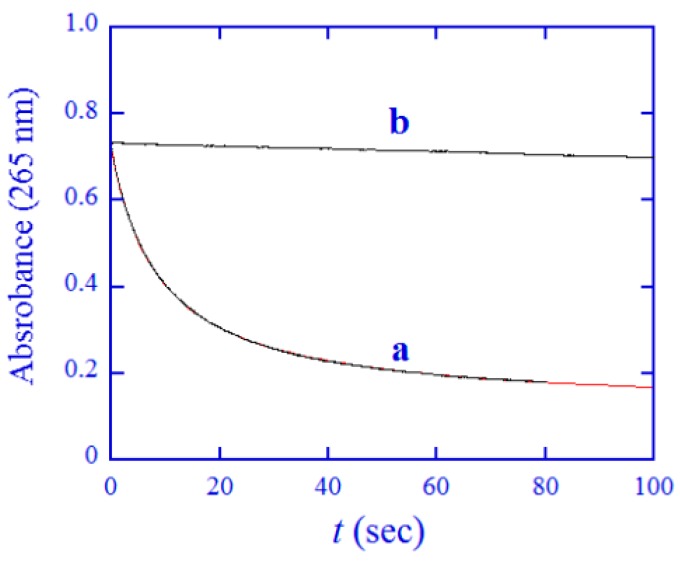
(**a**) Kinetic trace at 265 nm recorded in a phosphate buffer of pH 7.40, at 37.0 °C and *μ* = 1.0 M for the reaction between 0.050 mM ascorbate and 0.050 mM [Pt(cbdca)(NH_3_)_2_Cl_2_] (black line) and the simulated result by Equation (2) (red line). (**b**) Kinetic trace at 265 nm recorded for 0.050 mM ascorbate without the addition of the Pt(IV) complex.

The pseudo first-order conditions used for the reactions with thiols could not be used for the reaction with ascorbate, since the absorbance of ascorbate would overshadow that of the Pt(IV) prodrug in the wavelength region used. The redox reaction was thus studied under the conditions of [Pt(IV)] = [Asc]_tot_ = 0.10 mM in the pH 7.40 buffer. A typical kinetic trace recorded at 240 nm (early part of the reaction) is shown in Figure 4 (curve a), indicating that the redox reaction was fast. The kinetic trace was simulated by Equation (2), where *A*, *A*_0_, and *A*_∞_ denote the absorbance at time *t*, 0, and infinity, respectively, and *k*′ is the observed second-order rate constant [42].

*A* = (*A*_0_ − *A*_∞_)/(1 + *k*′[Pt(IV)]*t*) + *A*_∞_ (2)

The simulation resulted in an excellent fit (Figure 4), demonstrating that the redox reaction can be described by overall second-order kinetics, as observed for other reaction systems of Pt(IV) prodrugs [23]. Since ascorbate and dehydro-ascorbate are not stable in neutral buffers [43,44], a blank experiment was also run, in which a freshly prepared solution of ascorbate without addition of the Pt(IV) prodrug was used (curve b in Figure 4). That experiment showed that a slow degradation of ascorbate took place even if the solution was flushed by nitrogen gas and 2 mM EDTA was added. Consequently, stock solutions of ascorbate in pH 7.40 buffer were only used for less than 20 min in all experiments. The *k*′ values obtained from the simulations (also listed in Table 1 in the case of ascorbate) were not corrected for this slow degradation of ascorbate. Here, we need to point out that if Pt(IV) prodrugs are reduced slowly by ascorbate at a pH around 7 as reported previously [45,46,47], the accuracy of the reported redox rate constants should be considered with some caution, since they were not corrected for the degradation of ascorbate and the instability of dehydro-ascorbate. 

### 3.3. Rate Comparison and Biological Implications

The rate constants in Table 1 reveal the following reactivity order at physiological pH: Asc << Cys–Gly ~ Hcy < GSH < Cys. If the total concentrations of the reduced forms in human plasma (i.e. [Cys]_tot_ = 8.3–10.7 μM [22], [GSH]_tot_ = 2.0–5.1 μM [22], [Hcy]_tot_ = 0.17 –0.32 μM [22], [Cys-Gly]_tot_ = 2.0–2.9 μM [22], and [Asc]_tot_ = 4–150 μM [22,48]) are taken into account, the range of *k*_obsd_ for each reductant at 37.0 °C can be estimated, as well as the range of half-life (*t_½_*) for *cis,trans*-[Pt(cbdca)(NH_3_)_2_Cl_2_] at 37.0 °C for each reductant, if only that particular reductant is assumed to be present. These estimated ranges of *k*_obsd_ and *t_½_* are also given in Table 1. Several conclusions can be drawn from these half-life ranges: (1) Cys most probably will play a leading role in the reduction of the Pt(IV) prodrug, whereas Hcy will be much less important. (2) The ranges of half-lives of GSH, Cys–Gly, and Asc are overlapping and are not far from that of Cys, indicating that reductive activation by these three reductants might also take place. (3) In the reductive activation processes of the Pt(IV) prodrugs in human plasma, Asc is not predominant compared to the small thiols. (4) *cis,trans*-[Pt(cbdca)(NH_3_)_2_Cl_2_] has a very short lifetime in human plasma (seconds up to a minute) and is rapidly reduced to carboplatin. In such a short time, the Pt(IV) prodrug will have essentially no chance to enter cancerous cells, either actively or passively. Hence, the reduction of *cis,trans*-[Pt(cbdca)(NH_3_)_2_Cl_2_] to carboplatin will certainly take place extracellularly. 

When the reduction of *cis,trans*-[Pt(cbdca)(NH_3_)_2_Cl_2_] by these biomolecules is compared to those of ormaplatin ([Pt(dach)Cl_4_]) and the cisplatin prodrug *cis*-[Pt(NH_3_)_2_Cl_4_] [23], a reactivity order of *cis*-[Pt(NH_3_)_2_Cl_4_] > *cis,trans*-[Pt(cbdca)(NH_3_)_2_Cl_2_] > [Pt(dach)Cl_4_] is disclosed. However, the difference in reactivity is not large, indicating that the four equatorially coordinated ligands in these prodrugs have a limited influence on their reduction rates. The generally high reduction rate of *trans*-dichlorido-Pt(IV) anticancer prodrugs in human plasma, which is influenced slightly by the four equatorially coordinated ligands in the prodrugs, accounts for the fact that this type of anticancer prodrugs are not anymore considered in recent studies focused on the synthesis of Pt(IV) anticancer active compounds.

### 3.4. Reactivity of the Cys and GSH Species in the Reduction of cis,trans-[Pt(cbdca)(NH_3_)_2_Cl_2_]

**Figure 5:**
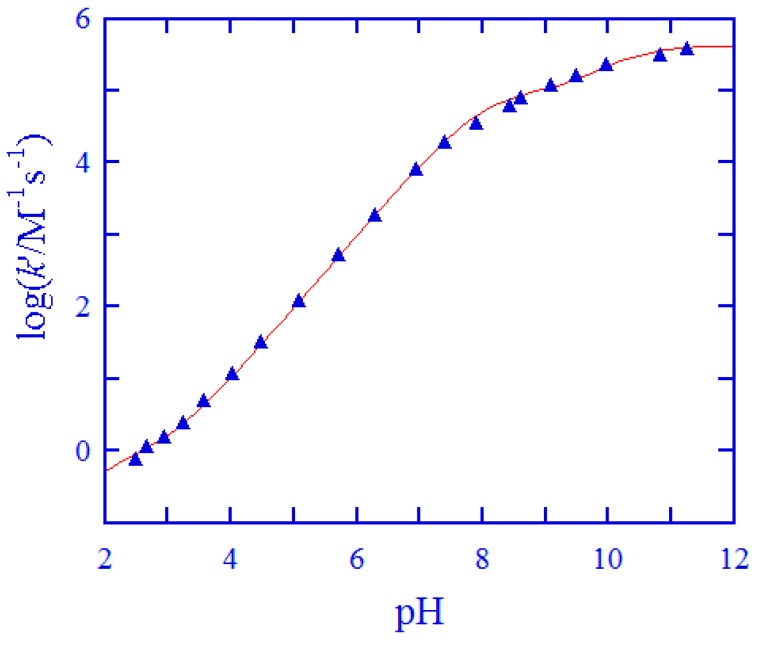
Second-order rate constants *k*′ as a function of pH at 25.0 °C and *μ* = 1.0 M for the reduction of *cis,trans*-[Pt(cbdca)(NH_3_)_2_Cl_2_] by Cys (data points). The solid curve was attained by fitting Equation (5) to the experimental data using a weighted nonlinear least-squares method.

**Figure 6:**
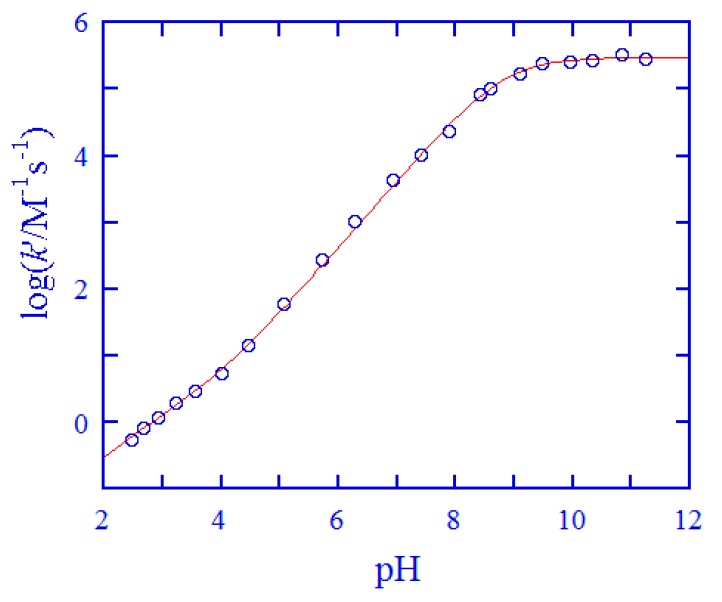
Second-order rate constants *k*′ as a function of pH at 25.0 °C and *μ* = 1.0 M for the reduction of *cis,trans*-[Pt(cbdca)(NH_3_)_2_Cl_2_] by GSH (data points). The solid curve was attained by fitting Equation (6) to the experimental data using a weighted nonlinear least-squares method.

**Figure 7:**
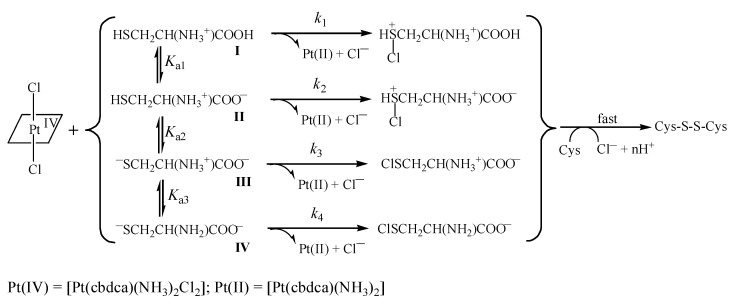
Reaction mechanism suggested for the reduction of [Pt(cbdca)(NH_3_)_2_Cl_2_] by Cys.

After ascertaining the importance of Cys and GSH in the reduction of *cis,trans*-[Pt(cbdca)(NH_3_)_2_Cl_2_], the redox reactions were investigated in a wide pH range for these two thiols. The kinetic characters, including overall second-order kinetics, a drastic increase of *k*′ with the increase of pH (cf. Table S1 in Supplementary Material), and the log*k*′ versus pH profiles shown in Figure 5 and Figure 6, were all similar to those observed for the reduction of ormaplatin by Cys [37] and GSH [49]. Thus, it was anticipated that similar reaction mechanisms should be valid also in the examined reaction systems. These are depicted in Figure 7 for the reaction of Cys and in Figure S3 in Supplementary Materials for the reaction of GSH. The reaction mechanisms involve all the protolytic species of Cys/GSH which attack concurrently one of the *trans*-dichloride ligands of the Pt(IV) prodrug, forming a bridge and then resulting in a Cl^+^ transfer from the Pt(IV) center to the attacking sulfur atoms and in the formation of chlorothiol and sulfenylchloride as transient species (cf. Figure 7 and Figure S7) [50,51,52,53]. These transient species will be trapped rapidly by excess of Cys/GSH, generating the oxidation products (cystine for Cys and the oxidized glutathione, i.e. GSSG for GSH). This type of electron transfer has been known for a long time [50,51,52,53] and it is strongly supported by a recent theoretical study [32]. Product analysis was carried out by mass spectrometry in the case Cys. The high-resolution mass spectra are shown in Figure S4 in Supplementary Material, and the peak assignments are given in the Figure legend. From the mass spectral analysis, formation of cystine and carboplatin was confirmed unambiguously.

Equation (3) is the rate expression derived from the reaction mechanism in Figure 7:

(3) $$-d\left[\mathrm{Pt}\left(\mathrm{IV}\right)\right]/\mathrm{dt}\;=\frac{k_{1}\alpha_{H}^{3}+k_{2}K_{a1}\alpha_{H}^{2}+k_{3}K_{a1}K_{a2}\alpha_{H}+k_{4}K_{a1}K_{a2}K_{a3}}{\alpha_{H}^{3}+K_{a1}\alpha_{H}^{2}+K_{a1}K_{a2}\alpha_{H}+K_{a1}K_{a2}K_{a3}}\left[\mathrm{Cys}\right]\mathrm{tot}\left[\mathrm{Pt}\left(\mathrm{IV}\right)\right]$$

In this equation, α_H_ denotes proton activity, exactly matching the pH measurements. A comparison of Equation (3) with Equation (1) gives:

(4) $$k{}^{\prime }=\frac{k_{1}\alpha_{H}^{3}+k_{2}K_{a1}\alpha_{H}^{2}+k_{3}K_{a1}K_{a2}\alpha_{H}+k_{4}K_{a1}K_{a2}K_{a3}}{\alpha_{H}^{3}+K_{a1}\alpha_{H}^{2}+K_{a1}K_{a2}\alpha_{H}+K_{a1}K_{a2}K_{a3}}$$

For L-cysteine, protolysis constants were reported to be p*K*_a1_ = 1.9, p*K*_a2_ = 8.07, and p*K*_a3_ = 9.95 at 25.0 °C and *μ* = 1.0 M [54]. Equation (4) was utilized to simulate the *k*′–pH data in Figure 5 by use of a weighted nonlinear least-squares routine [55]. In this simulation, the protolysis constants of Cys were employed as fixed values, whereas *k*_1_–*k*_4_ were treated as adjustable parameters. The simulation showed that *k*_1_ was too small to be estimated (or was indeterminate). Thus, the *k*_1_ term in Equation (4) could be neglected, resulting in Equation (5):

(5) $$k{}^{\prime }=\;\frac{k_{2}K_{a1}\alpha_{H}^{2}+k_{3}K_{a1}K_{a2}\alpha_{H}+k_{4}K_{a1}K_{a2}K_{a3}}{\alpha_{H}^{3}+K_{a1}\alpha_{H}^{2}+K_{a1}K_{a2}\alpha_{H}+K_{a1}K_{a2}K_{a3}}$$

The simulation of the *k*′–pH data in Figure 5 by Equation (5) afforded an excellent fit and provided the values of *k*_2_–*k*_4_ given in Table 2.

In analogy, Equation (6) is the rate expression (without the *k*_1_ term) deduced from the reaction mechanism of reduction of [Pt(cbdca)(NH_3_)_2_Cl_2_] by GSH [49]. Protolysis constants for GSH were reported at 25.0 °C and *μ* = 1.0 M [56]: p*K*_a1_ = 2, p*K*_a2_ = 3.35, p*K*_a3_ = 8.64, and p*K*_a4_ = 9.44. When the values of p*K*_a1_–p*K*_a4_ were used as direct inputs and *k*_2_–*k*_5_ were treated as tunable parameters, Equation (6) was applied to simulate the *k*′–pH data in Figure 6. A nice fit was obtained in this case also; the acquired values of *k*_2_–*k*_5_ from the simulation are listed in Table 2 as well.

(6) $$k{}^{\prime }=\;\frac{k_{2}K_{a1}\alpha_{H}^{3}+k_{3}K_{a1}K_{a2}\alpha_{H}^{2}+k_{4}K_{a1}K_{a2}K_{a3}\alpha_{H}+k_{5}K_{a1}K_{a2}K_{a3}K_{a4}}{\alpha_{H}^{4}+K_{a1}\alpha_{H}^{3}+K_{a1}K_{a2}\alpha_{H}^{2}+K_{a1}K_{a2}K_{a3}\alpha_{H}+K_{a1}K_{a2}K_{a3}K_{a4}}$$

The excellent agreement between experimental data and theoretical simulations shown in Figure 5 and Figure 6 bolsters the validity of the reaction mechanisms given in Figure 7 and Figure S3. By use of the obtained rate constants in Table 2 and the p*K*_a_ values for the various protolytic species [54,56], the reactivity of the various Cys and GSH species in the reduction of *cis,trans*-[Pt(cbdca)(NH_3_)_2_Cl_2_] was analyzed in terms of reactivity versus pH distribution diagrams [38,39]. These are shown in Figure S5 for Cys and in Figure S6 for GSH in Supplementary Material. The reactivity of the fully protonated species **I** was too small to be acquired with any certainty both for Cys and GSH; similar results have been obtained previously for the reduction of ormaplatin [37,49]. The human plasma has a physiological pH of about 7.4; at this pH, species **III** of Cys (^−^SCH_2_CH(NH_3_^+^)COO^−^) makes a 99% contribution to the total reactivity, even if it amounts to only 17.6% of the total population of Cys (cf. Figure 7 and Figure S5). In the case of GSH, species **IV** (^−^OOCCH(NH_3_^+^)CH_2_CH_2_CONHCH(CH_2_S^−^)CONH- CH_2_COO^−^) contributes with 98.6% of the total reactivity, although it only amounts to 5.4% to the total GSH population (cf. Figures S3 and S6). This analysis demonstrates unambiguously that species **III** of Cys and species **IV** of GSH are the exclusively predominant protolytic species in the reduction of *cis,trans*-[Pt(cbdca)(NH_3_)_2_Cl_2_] at physiological conditions.

## 4. Conclusions

The reductive activation of the carboplatin prodrug *cis,trans*-[Pt(cbdca)(NH_3_)_2_Cl_2_] by the predominant small-molecule reductants in human plasma was analyzed kinetically. Several major conclusions can be drawn from this analysis: (1) The lifetime of the prodrug is very short in human plasma, and the prodrug has essentially no time to enter cancerous cells before being reduced; certainly, the reduction takes place extracellularly. (2) *trans*-dichlorido-Pt(IV) prodrugs including ormaplatin, the cisplatin prodrug *cis*-[Pt(NH_3_)_2_Cl_4_], and *cis,trans*-[Pt(cbdca)(NH_3_)_2_Cl_2_] are all reduced too rapidly in human plasma to be able to enter cancerous cells. In addition, the difference in the reduction rates between these three prodrugs is not large. (3) l-Cys plays a leading role in the reduction process, followed by GSH and ascorbate, while Hcy has a limited role. (4) The proposed reaction mechanisms are very reasonable as demonstrated by the excellent curve fits between theoretical equations and experimental data in a wide pH range. (5) Species **III** of Cys and species **IV** of GSH are exclusively predominant in the reduction process in human plasma, as revealed by the reactivity versus pH distribution diagrams. These two species are anticipated to be of pivotal importance also in the reduction of other types of Pt(IV) prodrugs.

## Figures and Tables

**Table 1:** Summary of the observed second-order rate constants for the reduction of *cis,trans*-[Pt(cbdca)(NH_3_)_2_Cl_2_] by the predominant reductants in human plasma at pH 7.40 and *µ* = 1.0 M.

Reductant	*k*′/M^−1^s^−1^ (25.0 °C)	*k*′/M^−1^s^−1^ (37.0 °C)	*k*_obsd_/s^−1^ (37.0 °C) ^a^	*t*_½_/s (37.0 °C) ^a^
Cys	(1.93 ± 0.06) × 10^4^	(4.7 ± 0.2) × 10^4^	0.39–0.48	1.8–1.4
GSH	(1.01 ± 0.03) × 10^4^	(2.65 ± 0.09) × 10^4^	0.053–0.135	15–5
Hcy	(6.3 ± 0.2) × 10^3^	(1.70 ± 0.08) × 10^4^	0.0029–0.0054	240–127
Cys–Gly	(8.2 ± 0.2) × 10^3^	(1.57 ± 0.05) × 10^4^	0.031–0.046	22–15
Asc	(1.03 ± 0.04) × 10^3^	(2.2 ± 0.2) × 10^3^	0.0088–0.33	79–2.1

^a^ Estimated ranges of *k*_obsd_ and *t_½_* at the concentrations of reductants present in the human plasma.

**Table 2:** Rate constants obtained for the rate-determining steps in the reductions of *cis,trans*-[Pt(cbdca)(NH_3_)_2_Cl_2_] by Cys and GSH at 25.0 °C and *μ* = 1.0 M.

Thiol	*k*_m_	Value/M^−1^s^−1^
Cys	*k*_1_^a^	not obsd
	*k*_2_^a^	0.84 ± 0.09
	*k*_3_^a^	(9.7 ± 0.2) × 10^4^
	*k*_4_^a^	(4.2 ± 0.2) × 10^5^
GSH	*k*_1_^b^	not obsd
	*k*_2_^b^	0.45 ± 0.05
	*k*_3_^b^	3.0 ± 0.2
	*k*_4_^b^	(1.89 ± 0.08) × 10^5^
	*k*_5_^b^	(2.99 ± 0.09) × 10^5^

^a^ For definitions, see Figure 7. ^b^ For definitions, see Figure S3.

## Supplementary Materials

Supplementary Materials can be found at https://www.mdpi.com/1422-0067/20/22/5660/s1.
