# Supplementary material for: Investigations of the Kinetics and Mechanism of Reduction of a Carboplatin Pt(IV) Prodrug by the Major Small-Molecule Reductants in Human Plasma
Source: Int J Mol Sci. 2019 Nov 12;20(22):5660. doi: 10.3390/ijms20225660 (PMC6888404; doi:10.3390/ijms20225660)
Supplement: Supplementary file 1 [file ijms-20-05660-s001.pdf]

International Journal of *Molecular Sciences*

MDPI

**Supplementary Material**

# Investigations of the Kinetics and Mechanism of Reduction of a Carboplatin Pt(IV) Prodrug by the Major Small-Molecule Reductants in Human Plasma

One supporting table (Table S1) and six supporting figures (Figures S1-S6) are included in the Supplementary Material.

**Table S1.** Observed second-order rate constants *k*′ for the reduction of *cis,trans*-[Pt(cbdca)($NH_3$)$_2$$Cl_2$] by Cys and GSH as a function of pH at 25.0 °C and $\mu$ = 1.0 M.

| Thiol | pH | $k'/M^{-1}s^{-1}$ |
|---|---|---|
| Cys | 2.47 | 0.79 ± 0.02 |
| | 2.67 | 1.15 ± 0.03 |
| | 2.93 | 1.62 ± 0.04 |
| | 3.23 | 2.50 ± 0.06 |
| | 3.57 | 5.16 ± 0.15 |
| | 4.01 | 11.9 ± 0.2 |
| | 4.47 | 32.9 ± 0.7 |
| | 5.07 | 127 ± 3 |
| | 5.72 | 549 ± 9 |
| | 6.29 | (1.96 ± 0.06) x $10^3$ |
| | 6.94 | (8.2 ± 0.2) x $10^3$ |
| | 7.40 | (1.93 ± 0.06) x $10^4$ |
| | 7.90 | (3.70 ± 0.09) x $10^4$ |
| | 8.43 | (6.2 ± 0.1) x $10^4$ |
| | 8.60 | (8.0 ± 0.2) x $10^4$ |
| | 9.09 | (1.22 ± 0.03) x $10^5$ |
| | 9.48 | (1.66 ± 0.04) x $10^5$ |
| | 9.96 | (2.37 ± 0.08) x $10^5$ |
| | 10.83 | (3.22 ± 0.15) x $10^5$ |
| | 11.24 | (3.86± 0.16) x $10^5$ |
| GSH | 2.47 | 0.54 ± 0.02 |
| | 2.67 | 0.82 ± 0.02 |
| | 2.93 | 1.19 ± 0.03 |
| | 3.23 | 1.96 ± 0.04 |
| | 3.57 | 2.99 ± 0.06 |
| | 4.01 | 5.36 ± 0.15 |

| | |
|---|---|
| 4.47 | 14.4 ± 0.3 |
| 5.07 | 59.0 ± 1.5 |
| 5.72 | 272 ± 8 |
| 6.29 | (1.02 ± 0.03) x $10^3$ |
| 6.94 | (4.34 ± 0.09) x $10^3$ |
| 7.40 | (1.01 ± 0.03) x $10^4$ |
| 7.90 | (2.38 ± 0.06) x $10^4$ |
| 8.43 | (8.15 ± 0.19) x $10^4$ |
| 8.60 | (1.00 ± 0.03) x $10^5$ |
| 9.09 | (1.74 ± 0.04) x $10^5$ |
| 9.48 | (2.41 ± 0.06) x $10^5$ |
| 9.96 | (2.59 ± 0.08) x $10^5$ |
| 10.34 | (2.74 ± 0.08) x $10^5$ |
| 10.83 | (3.30 ± 0.08) x $10^5$ |
| 11.24 | (2.83 ± 0.08) x $10^5$ |

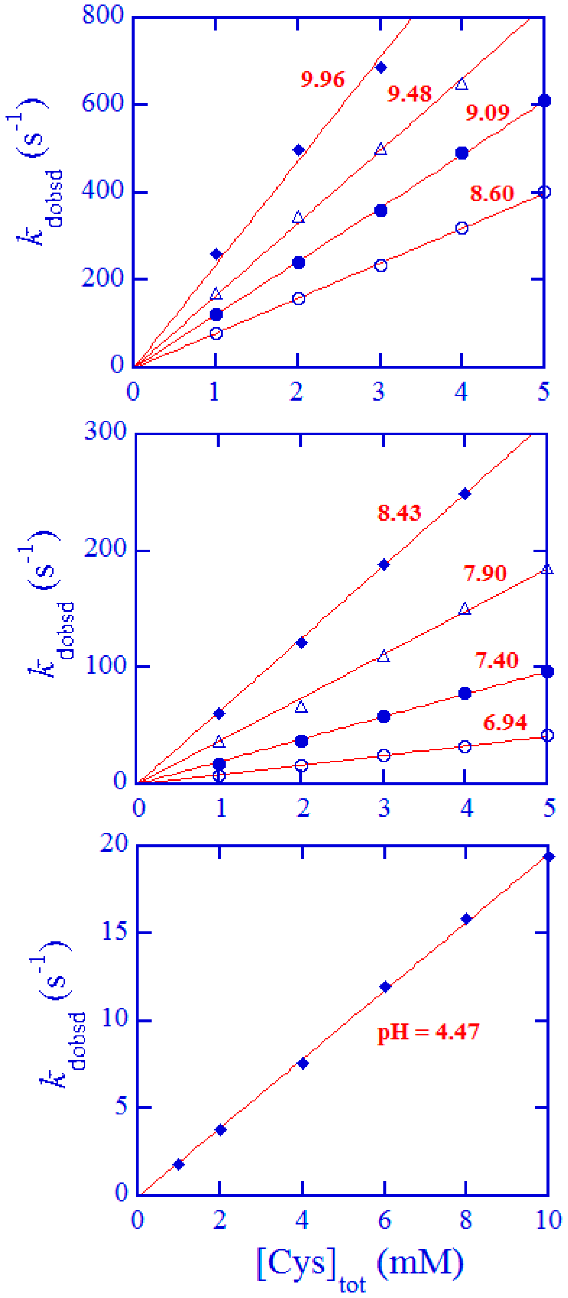


**Figure S1.** Plots of $k_{obsd}$ versus $[Cys]_{tot}$ for the reduction of *cis,trans*-$[Pt(cbdca)(NH_3)_2Cl_2]$ by Cys at 25.0 °C, $\mu$ = 1.0 M in buffer solutions at pH between 4.47 and 9.96 as denoted by the red numbers above each line.

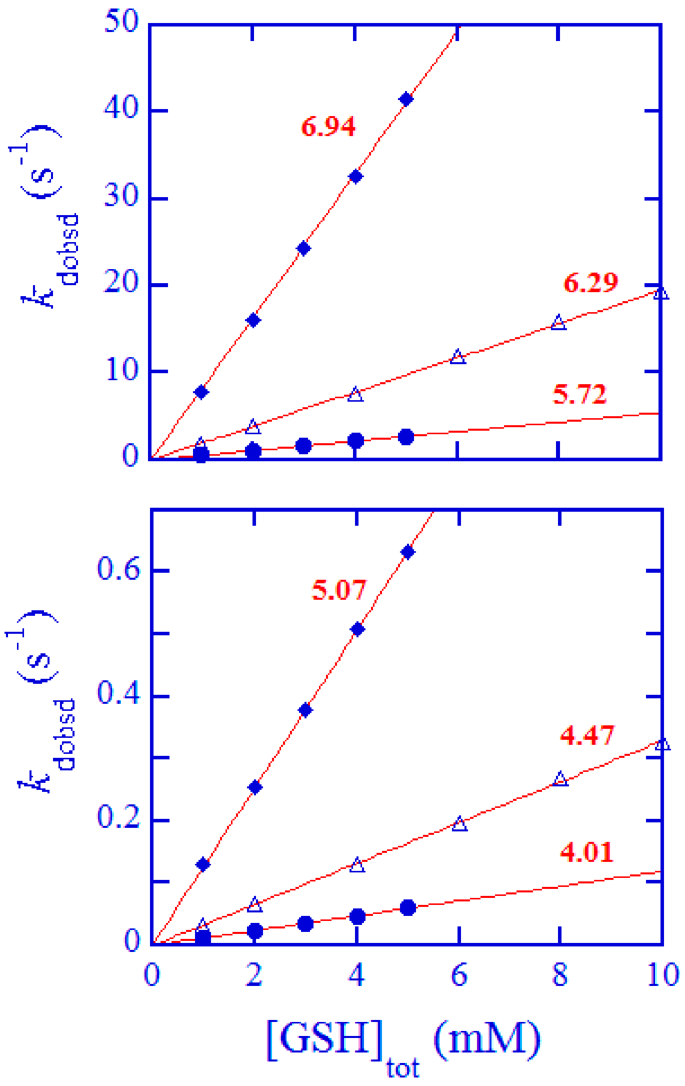


**Figure S2**. Plots of $k_{obsd}$ versus $[GSH]_{tot}$ for the reduction of *cis,trans*-[Pt(cbdca)($NH_3$)$_2$$Cl_2$] by GSH at 25.0 °C, $\mu$ = 1.0 M in buffer solutions at pH between 4.01 and 6.94 as denoted by the red numbers above each line.

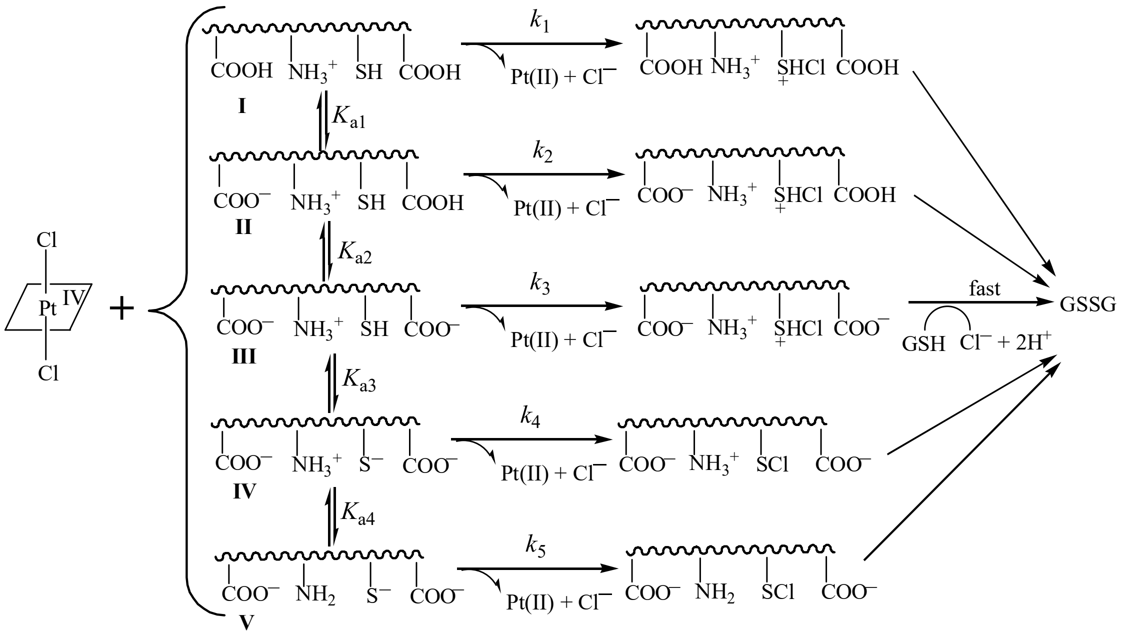


Pt(IV) = *cis,trans*-[Pt(cbdca)$(NH_3)_2Cl_2$]; Pt(II) = *cis*-[Pt(cbdca)$(NH_3)_2$]

**Figure S3.** Reaction mechanism proposed for the reduction of *cis,trans*-[Pt(cbdca)$(NH_3)_2Cl_2$] by GSH.

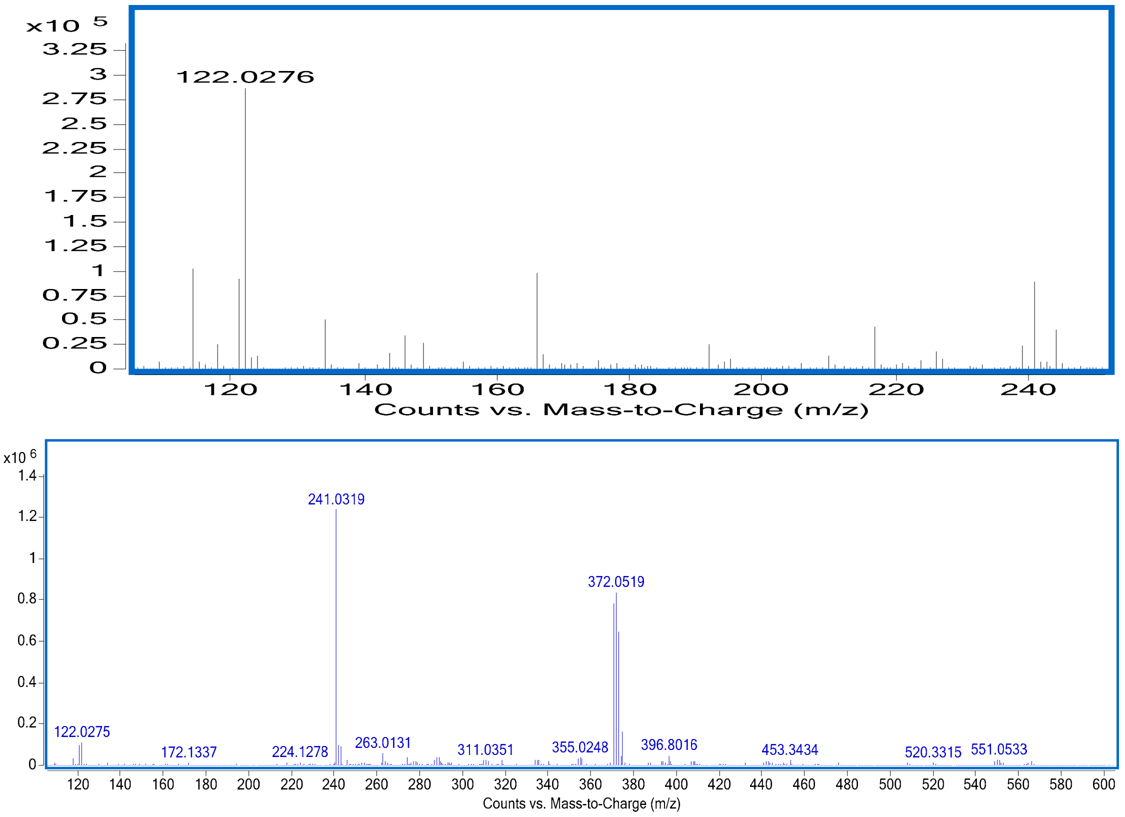


**Figure S4.** High-resolution mass spectra. (Top): 10 mM L-Cys in 10 mM HAc. (Bottom): A reaction mixture of 10 mM L-Cys with 1 mM *cis,trans*-[Pt(cbdca)$(NH_3)_2Cl_2$] in 10 mM HAc after a reaction time of 5 min.

**Peak assignments:** $m/z$ = 122.027 for Cys·$H^+$; $m/z$ = 241.03 for CysS-SCys·$H^+$; $m/z$ = 372.05 for carboplatin·$H^+$ (or *cis*-[Pt(cbdca)$(NH_3)_2$]·$H^+$).

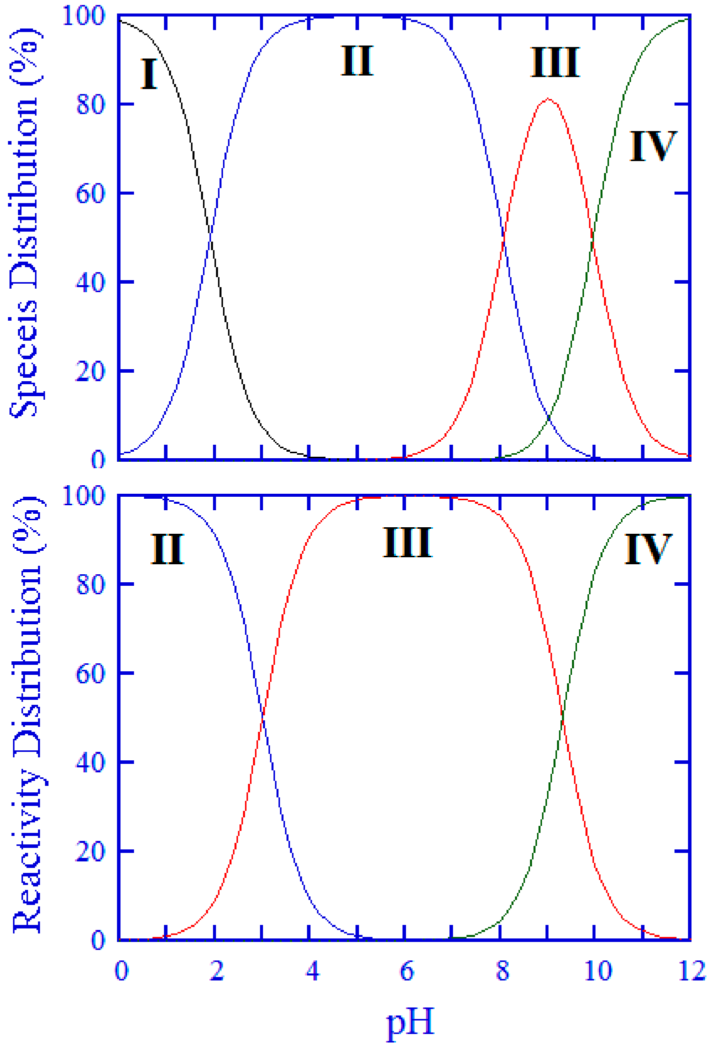


**Figure S5.** (Top): Distribution diagram for the various Cys protolytic species versus pH calculated from p$K_{a1}$ = 1.9, p$K_{a2}$ = 8.07, and p$K_{a3}$ = 9.95. (Bottom): Reactivity fraction of the Cys species in the reduction of *cis,trans*-[Pt(cbdca)($NH_3$)$_2$$Cl_2$] versus pH. The p$K_a$ values and the rate constants in Table 2 were utilized to generate this diagram. Structures of the Cys species **I** – **IV** are given in Figure 7 in the main text.

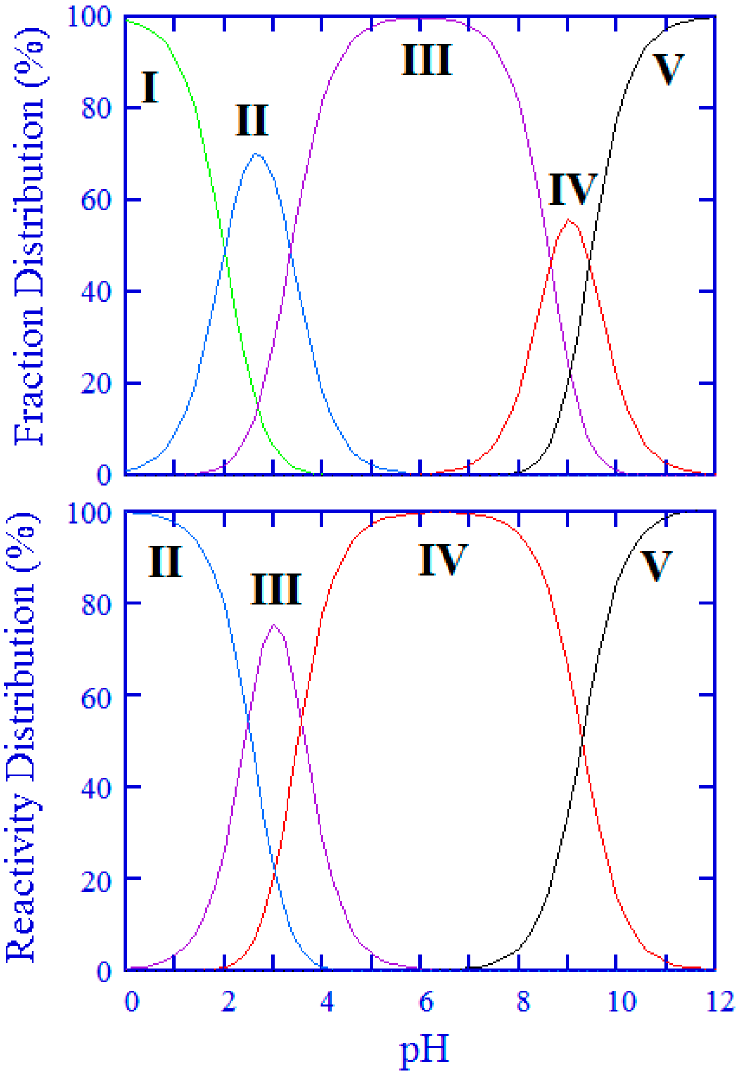


**Figure S6.** (Top): Distribution diagram for the various GSH protolytic species versus pH calculated from $pK_{a1} = 2$, $pK_{a2} = 3.35$, $pK_{a3} = 8.64$ and $pK_{a4} = 9.44$. (Bottom): Reactivity fraction of the GSH species in the reduction of *cis,trans*-[Pt(cbdca)$(NH_3)_2Cl_2$] versus pH. The $pK_a$ values and the rate constants in Table 2 were utilized to generate this diagram. Structures of the GSH species **I** – **V** are given in Figure S3.

© 2019 by the authors. Licensee MDPI, Basel, Switzerland. This article is an open access article distributed under the terms and conditions of the Creative Commons Attribution (CC BY) license (http://creativecommons.org/licenses/by/4.0/).
